# CDC Grand Rounds: National Amyotrophic Lateral Sclerosis (ALS) Registry Impact, Challenges, and Future Directions

**DOI:** 10.15585/mmwr.mm6650a3

**Published:** 2017-12-22

**Authors:** Paul Mehta, D. Kevin Horton, Edward J. Kasarskis, Ed Tessaro, M. Shira Eisenberg, Susan Laird, John Iskander

**Affiliations:** ^1^Environmental Health and Surveillance Branch, Division of Toxicology and Human Health Sciences, Agency for Toxic Substances and Disease Registry, CDC; ^2^Cynthia Shaw Crispen Chair, ALS Research, Department of Neurology, Lexington, University of Kentucky; ^3^person living with ALS; ^4^Office of the Associate Director for Science, CDC; ^5^Office of the Associate Director for Communication Science, CDC.

Amyotrophic lateral sclerosis (ALS), commonly known as Lou Gehrig’s disease, is a rapidly progressive fatal neurologic disease. Currently, there is no cure for ALS and the available treatments only extend life by an average of a few months. The majority of ALS patients die within 2–5 years of diagnosis, though survival time varies depending on disease progression (*1*,*2*). For approximately 10% of patients, ALS is familial, meaning it and has a genetic component; the remaining 90% have sporadic ALS, where etiology is unknown, but might be linked to environmental factors such as chemical exposures (e.g., heavy metals, pesticides) and occupational history (*3*).

Like many other noncommunicable conditions, ALS is a nonnotifiable disease in the United States; therefore, the federal government lacks reliable incidence and prevalence estimates for the United States. During October 2008, Congress passed the ALS Registry Act (*4*), directing CDC and its sister agency, the Agency for Toxic Substances and Disease Registry, to create a population-based ALS registry for the United States. The main objectives of the National ALS Registry, which was launched in October 2010, are to describe the national incidence and prevalence of ALS; describe the demographics of persons living with ALS; and examine risk factors for the disease (*4*,*5*). During January 2017, the Registry launched the National ALS Biorepository, which aims to promote research in areas including biomarkers, genetics, and environmental exposures to heavy metals or organophosphates (*6*,*7*).

## ALS Registry and Biorepository Methods and Impact

Because ALS is a nonnotifiable condition, the National ALS Registry uses a novel two-pronged approach for identifying cases in the United States (*5*) including searching national administrative databases and self-identification. The first approach applies a pilot-tested algorithm to large national databases (e.g., Medicare, Veterans Health Administration) to identify cases (*5*,*8*). The algorithm helps classify individual persons as having actual, potential, or non-ALS cases using variables including the *International Classification of Diseases – Ninth Revision* (ICD-9) diagnostic code for ALS, frequency of visits to neurologists, and use of prescription drugs (e.g., Rilutek) (*8*). Patients with ALS are added directly to the Registry, while those considered noncases are not. Potential ALS patients are not added to the Registry, but are retained until subsequent years of administrative data are available to be able to make a determination (*8*). The second approach uses a secure web portal to allow persons with ALS to self-identify (*8*). ALS patients answer a series of online validation questions (e.g., has a doctor ever diagnosed you with ALS?). Their responses to these questions determine whether they are considered actual ALS cases (*8*). In addition, this web portal approach allows ALS enrollees to take brief online risk factor surveys (e.g., occupational history, residential history, history of head trauma) that will allow scientists to learn more about the possible causes of ALS (*8*). Cases from both approaches are then merged and deduplicated so that cases are not counted multiple times (*8*) (Figure).

**FIGURE:**
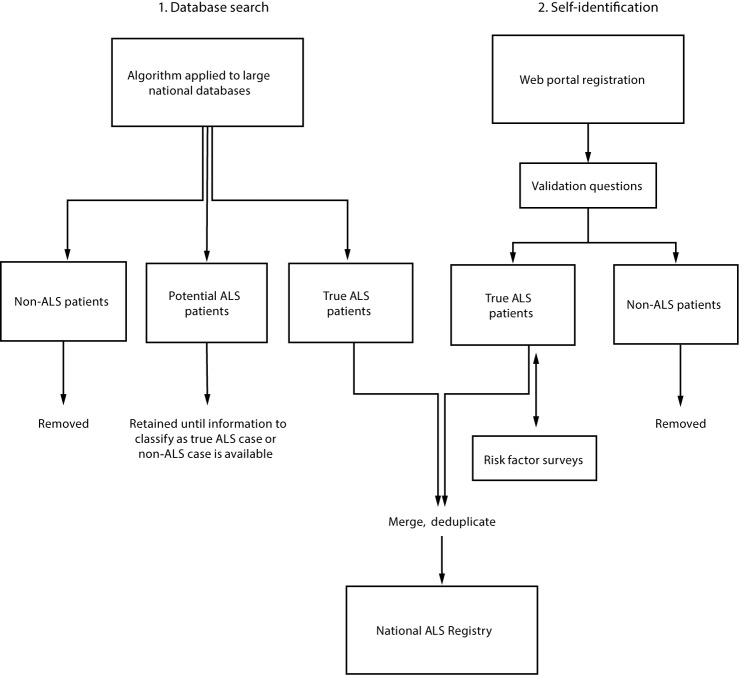
Methodology* for identification of amyotrophic lateral sclerosis (ALS) cases for inclusion in the National ALS Registry — United States, 2013 * *International Classification of Diseases, Ninth Revision* (ICD-9) code, frequency of neurology visits, prescription drug use.

The National ALS Biorepository is part of the Registry; therefore, patients must enroll in the Registry to donate specimens (*6*). The Registry conducted a multiyear pilot study to determine the feasibility of the Biorepository (*6*). A group of external subject matter experts provided direction and deemed the Biorepository to be feasible, and it was launched in January 2017 (*6*). The Biorepository has a geographically representative sample collection scheme, that is, not all samples will come from one part of country, but are distributed based on population density (*7*). There are two components of the Biorepository: an in-home collection and a postmortem collection. The in-home collection consists of samples of blood, urine, and saliva from ALS patients, with an annual goal of 300 samples. The postmortem collection, consisting of samples of bone, brain, spinal cord, cerebrospinal fluid, and muscle targets 10 collections each year. The pre- and postmortem collections will seek to expand knowledge on ALS biomarkers, genetics, and ultimately, etiology. The Biorepository is unique in that the samples collected are not previously used or left over from another study. In addition, these samples will be matched with the Registry’s survey data as well as a Global Unique Identifier (for those patients who elect to have a global unique identifier generated), which will allow researchers to track the progress of patients in multiple studies securely and anonymously. When researchers request samples, they can receive, in addition to the samples, linked risk factor data such as demographics, occupation, and military service history (*7*). Lastly, the National ALS Biorepository will facilitate ALS research on etiologies and possible treatments.

## ALS Prevalence and Risk Factors

In 2013, the most current year for which data are available, the Registry identified almost 16,000 cases of ALS, corresponding to a prevalence of five cases per 100,000 population in the United States (*9*). As with any surveillance system for a disease that is nonnotifiable, it is impossible to capture all cases of ALS through the Registry. For example, there are currently no linkages to private insurance systems such as health maintenance organizations, where potential ALS patients might seek diagnosis or treatment.

ALS disproportionately affects whites, males, and persons aged 60–69 years (*9*); the reasons for the increased incidence among whites and males is unknown (*9*). Military veterans, particularly men, are at higher risk for developing ALS than are those who have not served (*10*). Veterans who served in the first Gulf War were twice as likely to develop ALS as were veterans who served during the same period but were not deployed to the Gulf (*11*). The reason for the increased risk among veterans is not known, but it might be related to selective environmental exposures (*9*,*10*).

Participation by athletes in certain sports, specifically American football, has purportedly been associated with an increased risk of developing ALS; several high-profile diagnoses in professional football players have also brought increased attention to ALS (*12*). Currently, it is unknown if football players might be at a greater risk for ALS than the general population; however, some research indicates it might be related to experiencing repeated concussions, or that ALS could be confused with a different condition such as chronic traumatic encephalopathy (*12*). More research is needed to investigate etiology of ALS and to learn more about the pathophysiology.

 ALS incidence is stable; however, the prevalence slowly continues to increase (*13*). Proposed reasons for the increase in prevalence includes comprehensive health care that allows patients to live longer, and large ALS clinics that provide patients with neurologic and nursing care, dietary support, and physical therapy care in one setting (*13*). However, not all ALS patients have access to large multidisciplinary ALS clinics, and those living in rural areas still tend to see their local primary care physician or neurologist (*13*,*14*).

## Challenges for Research, Drug Development, and Patient Care

The onset of ALS is insidious. Patients might experience weakness in an upper or lower limb or difficulty speaking or swallowing, with bulbar onset disease. No definitive blood, cerebrospinal fluid, or imaging biomarkers for ALS have been identified yet; thus, ALS is often a diagnosis of exclusion, typically made after other diseases have been ruled out (*15*). As a result, approximately 9–12 months might elapse during the onset of new progressive weakness and a definitive diagnosis. This time window, essentially one quarter of an ALS patient’s remaining lifespan, is a lost opportunity for developing drugs aimed at stopping the degeneration and death of motor neurons.

Researchers can measure and monitor ALS progression and the effectiveness of drugs in clinical trials using self-rating of function with the ALS Functional Rating Scale or quantitative measures of muscle power, including pulmonary function tests (e.g., percentage of forced vital capacity, maximum inspiratory pressure, sniff nasal pressure), measurement of walking speed, and isometric muscle power (*16*). However, disease progression varies widely among patients. Certain functions can remain normal including bladder and bowel control, eye movements, and awareness (*15*). Unlike other progressive neurologic conditions such as Alzheimer’s disease, cognition and largely memory remain intact for the vast majority of ALS patients; however, new research suggests that frontotemporal dementia may be affecting more ALS patients than previously thought (*17*).

Barriers to progress in identifying the etiology, means of prevention, and cure of ALS remain formidable. An estimated 50%–70% of motor neurons are no longer functional when patients with clinical signs and symptoms come to medical attention (*15*). Therefore, clinical trials that enroll ALS patients use drugs that can only attempt to slow disease progression. At this time, there are no identified therapeutics that stop or reverse the death of these motor neurons (*15*). Other barriers include the large number of patients required for sufficiently powered clinical trials and the costs of trials.

## Living with ALS: A Patient’s Perspective

A patient with ALS has written, “ALS patients can have a zeal for life rare among patients with other diseases. Shorter life expectancy often spurs patient with ALS to make life experiences and relationships deeper. It is helpful to understand the concept that ‘everyone has a wheel chair,’ and that no one avoids life’s crises forever.”

Organizations exist with the mission to defeat ALS through research, and provide support for the thousands of persons living with the disease in the United States. Such groups include the ALS Association, the Muscular Dystrophy Association, and the Les Turner ALS Foundation. However, more support for research is needed. Even with continued support from private donors, foundations, and institutions, rare diseases (those with <200,000 cases diagnosed nationwide)* like ALS still face barriers to research funding and treatment development.

The financial consequences of ALS after diagnosis can also be crippling, and go well beyond typical loss of income (*18*). Living with ALS becomes cost-prohibitive for a majority of patients (*18*). Some accommodations, including home conversion; a power wheelchair; and a van with ramp, lifts, and tech-assist devices can cost from $100,000 to $150,000, adding considerable stress to families already dealing with the diagnosis (*18*). The fear that family savings, retirement, mortgages, and educational funds are at risk, often provokes further health complications (*18*).

Development of a strong doctor-patient alliance can balance honest, diagnostic, and prognostic communications with messages that promote purpose, hope, and quality of life for patients with ALS. After the diagnosis, there is a great need to counsel patients in an affirmative way to accept the reality of the disease. Currently, this type of family counseling is rarely included in the ALS multispecialty clinic setting. Much can be done to help patients cope and see firsthand the optimism of new research, clinical trial enrollment, technology-based solutions, and self-determination techniques. Reluctance to spend time discussing these positive aspects for fear of creating false hope might result in a missed therapeutic opportunity.

## The National ALS Registry as a Model for 21st Century Surveillance

Whereas understanding the epidemiology of ALS is one of the main objectives of the National ALS Registry, the Registry also conducts other vital activities to help both patients and researchers learn more about the disease.

The Registry funds external research to help the ALS community learn more about potential ALS etiology and risk factors. To date, the Registry has funded 13 research projects including Large-Scale Genome-Wide Association Studies of ALS, gene-environment interaction studies, antecedent medical conditions, and environmental risk factors for ALS.

Importantly, the Registry is used to recruit enrollees into clinical trials and epidemiologic studies. The Registry speeds up difficult and costly clinical trial recruitment time, increases study sample size, and helps achieve racial, ethnic, and geographic diversity. The Registry’s services are provided free to researchers (*9*). To date, the Registry has helped scientists in the public and private sectors recruit hundreds of patients into over 30 research studies.

The National ALS Registry is the first and only population-based ALS registry for the United States that is quantifying the epidemiology of the disease (*8*). The Registry is a critical tool in building the evidence to describe the ALS experience in the United States, provide epidemiologic data and biospecimens to scientists, and discover the etiology and risk factors for ALS.

## References

[1] Miller RG, Jackson CE, Kasarskis EJ, ; Quality Standards Subcommittee of the American Academy of Neurology. Practice parameter update: the care of the patient with amyotrophic lateral sclerosis: drug, nutritional, and respiratory therapies (an evidence-based review): report of the Quality Standards Subcommittee of the American Academy of Neurology. Neurology 2009;73:1218–26. 10.1212/WNL.0b013e3181bc0141 19822872 PMC2764727

[2] Miller RG, Jackson CE, Kasarskis EJ, ; Quality Standards Subcommittee of the American Academy of Neurology. Practice parameter update: the care of the patient with amyotrophic lateral sclerosis: multidisciplinary care, symptom management, and cognitive/behavioral impairment (an evidence-based review): report of the Quality Standards Subcommittee of the American Academy of Neurology. Neurology 2009;73:1227–33. 10.1212/WNL.0b013e3181bc01a4 19822873 PMC2764728

[3] Bettencourt C, Houlden H. Exome sequencing uncovers hidden pathways in familial and sporadic ALS. Nat Neurosci 2015;18:611–3. 10.1038/nn.4012 25919956

[4] US Public Health Service. ALS Registry Act. Washington, DC: 110th Congress. Public Law 2008;122 Stat 4047:110–373.

[5] Bryan L, Kaye W, Antao V, Mehta P, Muravov O, Horton DK. Preliminary results of national amyotrophic lateral sclerosis (ALS) registry risk factor survey data. PLoS One 2016;11:e0153683. 10.1371/journal.pone.0153683 27124833 PMC4849726

[6] World Federation of Neurology Research Group on Motor Neuron Diseases. Theme 8 Epidemiology. Amyotrophic lateral sclerosis and frontotemporal degeneration. World Federation of Neurology Research Group on Motor Neuron Diseases; 2015. http://www.tandfonline.com/doi/abs/10.3109/21678421.2015.1098813

[7] Horton DK, Kaye W, Wagner L. Integrating a biorepository into the national amyotrophic lateral sclerosis registry. J Environ Health 2016;79:38–40. 28935999 PMC5603321

[8] Horton DK, Mehta P, Antao VC. Quantifying a nonnotifiable disease in the United States: the National Amyotrophic Lateral Sclerosis Registry model. JAMA 2014;312:1097–8. 25057819 10.1001/jama.2014.9799 PMC4550214

[9] Mehta P, Kaye W, Bryan L, Prevalence of amyotrophic lateral sclerosis — United States, 2012–2013. MMWR Surveill Summ 2016;65:1–12. 10.15585/mmwr.ss6508a1 27490513

[10] Oskarsson B, Horton DK, Mitsumoto H. Potential environmental factors in amyotrophic lateral sclerosis. Neurol Clin 2015;33:877–88. 10.1016/j.ncl.2015.07.009 26515627 PMC4646848

[11] Weisskopf MG, O’Reilly EJ, McCullough ML, Prospective study of military service and mortality from ALS. Neurology 2005;64:32–7. 10.1212/01.WNL.0000148649.17706.D9 15642900

[12] Lehman EJ, Hein MJ, Baron SL, Gersic CM. Neurodegenerative causes of death among retired national football league players. Neurology 2012;79:1970–4. 10.1212/WNL.0b013e31826daf50 22955124 PMC4098841

[13] Martin S, Trevor-Jones E, Khan S, The benefit of evolving multidisciplinary care in ALS: a diagnostic cohort survival comparison. Amyotroph Lateral Scler Frontotemporal Degener 2017;18:569–75. 10.1080/21678421.2017.1349151 28719997

[14] Wagner L, Rechtman L, Jordan H, State and metropolitan area-based amyotrophic lateral sclerosis (ALS) surveillance. Amyotroph Lateral Scler Frontotemporal Degener 2015;17:128–34. 10.3109/21678421.2015.1074699 26399278 PMC4732418

[15] Rowland LP, Shneider NA. Amyotrophic lateral sclerosis. N Engl J Med 2001;344:1688–700. 10.1056/NEJM200105313442207 11386269

[16] Andres PL, Allred MP, Stephens HE, Fixed dynamometry is more sensitive than vital capacity or ALS rating scale. Muscle Nerve 2017;56:710–5. 10.1002/mus.25586 28120413

[17] Strong MJ, Grace GM, Freedman M, Consensus criteria for the diagnosis of frontotemporal cognitive and behavioural syndromes in amyotrophic lateral sclerosis. Amyotroph Lateral Scler 2009;10:131–46. 10.1080/17482960802654364 19462523

[18] Obermann M, Lyon M. Financial cost of amyotrophic lateral sclerosis: a case study. Amyotroph Lateral Scler Frontotemporal Degener 2015;16:54–7. 10.3109/21678421.2014.951946 25245119

