# *Erratum*: Vol. 66, No. 50

**DOI:** 10.15585/mmwr.mm6702a8

**Published:** 2018-01-19

In the report “CDC Grand Rounds: National Amyotrophic Lateral Sclerosis (ALS) Registry Impact, Challenges, and Future Directions,” on page 1382, the list of author affiliations should have read as follows: 

^1^Environmental Health and Surveillance Branch, Division of Toxicology and Human Health Sciences, Agency for Toxic Substances and Disease Registry, CDC; ^2^Cynthia Shaw Crispen Chair, ALS Research, Department of Neurology, Lexington, University of Kentucky; ^3^person living with ALS; ^4^Office of the Associate Director for Science, CDC; **^5^Office of the Associate Director for Communication, CDC**.

